# Efficacy and safety of camrelizumab combined with albumin-bound paclitaxel as third- or later-line regimen in patients with advanced non-small cell lung cancer

**DOI:** 10.3389/fimmu.2023.1278573

**Published:** 2023-12-06

**Authors:** Jianfeng Zhu, Yanyan Yu, Jiaqi Mei, Shiyao Chen, Jiufei Li, Sicong Jiang

**Affiliations:** ^1^ Department of Thoracic Surgery, Jiangxi Province Cancer Hospital, Nanchang, Jiangxi, China; ^2^ Department of Operation Room, Jiangxi Province Cancer hospital, Nanchang, Jiangxi, China; ^3^ College of Innovation and Entrepreneurship, The First Clinical Medical College of Nanchang University, Nanchang, Jiangxi, China; ^4^ Department of Clinical Medicine, Jiangxi Institute of Applied Science and Technology, Nanchang, Jiangxi, China; ^5^ Department of Thoracic Surgery, The Affiliated Ganzhou Hospital of Nanchang University, Ganzhou, Jiangxi, China; ^6^ Division of Thoracic and Endocrine Surgery, University Hospitals and University of Geneva, Geneva, Switzerland

**Keywords:** non-small cell lung cancer, advanced, camrelizumab, albumin-bound paclitaxel, third- or later-line treatment

## Abstract

**Background:**

The clinical efficacy and safety of camrelizumab as a third- or later-line regimen in patients with advanced non-small cell lung cancer (NSCLC) have not been determined in large clinical trials.

**Objective:**

This study aimed to evaluate the clinical efficacy and safety of camrelizumab in combination with albumin-bound paclitaxel as a third- or later-line treatment for patients with advanced NSCLC.

**Methods:**

A total of 257 patients with advanced NSCLC who were histopathologically confirmed and failed in clinical second-line therapy regimens at Jiangxi Province Cancer hospital from January 2018 to December 2021 were retrospectively selected. Patients with advanced NSCLC were divided into the single treatment group (STG) of camrelizumab, and the combined treatment group (CTG) of camrelizumab in combination with albumin-bound paclitaxel according to the treatment regimen. The primary outcomes of interest were clinical efficacy[objective response rate (ORR) and disease control rate (DCR)], progression-free survival (PFS), and overall survival (OS). Survival data were analyzed using the Kaplan-Meier method, and the log-rank test was performed. Additionally, Cox proportional hazard regression was used to analyze the correlation of prognosis and baseline characteristics between subgroups, to identify the potential independent risk factors for PFS and OS. Furthermore, the occurrence of side effects was assessed according to the Common Terminology Criteria for Adverse Events (CTCAE 4.03).

**Results:**

Of the 257 patients with advanced NSCLC included in the research, 135 patients received camrelizumab, and 122 patients received camrelizumab plus albumin-bound paclitaxel. The ORR of CTG and STG was 59.84% and 50.38%, and the DCR was 77.05% and 65.93%, respectively. The median PFS in CTG was higher than that in the STG (5.27 vs. 3.57 months, P = 0.0074), and the median OS was longer (7.09 vs. 6.47 months, P < 0.01). The lines of treatment, metastases, and PD-L1 expression levels were independent risk factors for the mPFS and mOS of patients with advanced NSCLC. The occurrence of adverse events was similar between camrelizumab and camrelizumab plus albumin-bound paclitaxel groups.

**Conclusion:**

Camrelizumab combined with albumin-bound paclitaxel as the third- or later-line regimen greatly prolonged PFS and OS of advanced NSCLC patients. A prospective clinical trial is warranted.

## Introduction

Lung cancer is the leading malignancy in terms of incidence and mortality worldwide, with an incidence rate of 11.6% and a mortality rate of 18.4% (1). Non-small cell lung cancer (NSCLC) is a common sub-type of lung cancer (85%). Recently, diagnosis and therapy of malignancy have made great advances, but NSCLC patients are mostly in the advanced stage when diagnosed due to the lack of early clinical symptoms, therefore losing the surgical opportunity (2). In addition, patients with NSCLC could develop metastases even at an early stage (3). Therefore, the 5-year survival rate of NSCLC patients is low at 6% to 32% (1, 4).

The platinum-based drugs, as the basic chemotherapy, have been widely used in patients with advanced NSCLC for decades, but its efficacy is unsatisfactory (5). Luckily, with the application of molecularly targeted drugs, the survival rate of advanced NSCLC patients with related driver gene mutations has significantly improved. However, the rate of driver gene mutations in advanced NSCLC is 20%-30%, which suggests that the majority of advanced NSCLC patients could not benefit from the targeted therapy to achieve longer survival. Hence, improving treatment methods and prolonging survival time is crucial for advanced NSCLC patients.

Immunotherapy has revolutionized the clinical treatment of malignancy. Immune checkpoint inhibitors (ICIs) targeting the programmed death 1 (PD-1) axis plus chemotherapy have shown a better survival rate than those in chemotherapy monotherapy (6). Camrelizumab is a humanized, high-affinity IgG4-kappa monoclonal antibody against programmed cell death (PD-1), and has been applied in many clinical trials. CameL’s study showed that camrelizumab plus carboplatin and pemetrexed prolonged PFS in non-squamous NSCLC patients (11.3 months) with a mOS of 27.9 months (7). In addition, camrelizumab combined with chemotherapy as the second-line regimen significantly extended PFS (9.67 months vs. 6.87 months) and OS (10.89 months vs. 7.95 months) in advanced NSCLC (8). Although numerous clinical studies are focusing on the clinical efficacy of camrelizumab in advanced NSCLC, no large clinical trials have been reported on the clinical efficacy of camrelizumab as a third- or later-line regimen in advanced NSCLC patients.

In this study, we retrospectively recruited advanced NSCLC patients with a third- or later-line regimen that uses camrelizumab monotherapy or camrelizumab plus albumin-bound paclitaxel in Jiangxi Province Cancer hospital from January 2018 to December 2021. The aim was to investigate the clinical efficacy of camrelizumab plus albumin-bound paclitaxel as a third- or later-line regimen for advanced NSCLC patients, to provide a novel reference for advanced NSCLC patients’ treatment.

## Materials and methods

### Study design

We retrospectively analyzed the clinical data of histopathologically confirmed patients with advanced (IIIB-IV) NSCLC who failed in clinical second-line chemotherapy regimens at our hospital from January 2018 to December 2021. Patients who only received camrelizumab regimen were included in the single treatment group (STG) and those treated with added albumin-bound paclitaxel were classified into the combined treatment group (CTG). The Declaration of Helsinki was followed in the conduct of this study. The Ethics Committee of Jiangxi Province Cancer hospital approved the study protocol. To maintain privacy, no personally identifiable information was provided. Informed consent from the study participants was waived according to national legislation and institutional standards.

### Patient eligibility

Patients aged 18 to 75 years were eligible for inclusion in this study. The inclusion criteria were as follows: (1) pathologically confirmed diagnosis of advanced (stage IIIB-IV) NSCLC; (2) presence of at least one measurable lesion according to Response Evaluation Criteria in Solid Tumors (version 1.1); (3) failure of clinical second-line chemotherapy regimens; (4) Eastern Cooperative Oncology Group (ECOG) performance status of 0 or 1; (5) absence of driver gene mutations, such as EGFR, anaplastic lymphoma kinase (ALK), ROS, et al., or resistance and intolerance after the targeted drugs treatment; (6) expected survival time over than 3 months; (7) adequate hematologic, hepatic, and renal function.

Exclusion criteria were as follows: (1)psychiatric disorders, enabling unable to cooperate with treatment; (2) severe organ insufficiency; (3) not receiving Camrelizumab; (4) lack of a large number of baseline information, or data for efficacy assessment; (6) receiving other immunotherapy.

### Treatment regimen

All patients were treated with camrelizumab according to the following regimen: camrelizumab 200 mg Q3W was administered intravenously until disease progression, intolerable toxicity, or death. Patients in the STG were camrelizumab monotherapy. Patients in the CTG were treated intravenously with added albumin-bound paclitaxel, 260mg/m^2^ Q3W until disease progression, intolerable toxicity, or death. 3 weeks were defined as a treatment cycle.

### Efficacy and toxicity evaluation

The response was defined as the best-observed response after at least three cycles of the camrelizumab or camrelizumab plus albumin-bound paclitaxel regimen. All patients were evaluated for efficacy after the treatment, and computed tomography (CT) scans were performed every two cycles to detect changes in the lesions. The efficacy response was evaluated by complete remission (CR), partial remission (PR), stable disease (SD), and progression of disease (PD) according to the Response Evaluation Criteria of Solid Tumors (RECIST) standard. Additionally, objective response rate (ORR) and disease control rate (DCR) was calculated, in which ORR = (number of CR cases + number of PR cases)/number of evaluable patients × 100%, and DCR = (number of CR cases + number of PR cases + number of SD cases)/number of evaluable patients × 100%. Progression-free survival (PFS) was defined as the time from the start of treatment to disease progression or death. OS was defined as the time from the start of treatment to death for any reason.

The toxicity assessment is based on the Common Terminology Criteria for Adverse Events (CTCAE 4.03).

### Statistical analysis

Statistical software SPSS 22.0 (IBM Software, Armonk, NY, USA) was applied for data processing. Differences in measured data, expressed as mean ± standard error (SE), were compared using the student’s t-test. Comparisons of counting data, expressed as percentages, were performed using the chi-square test or Fisher’s exact test. The Kaplan-Meier method was used to estimate PFS and OS, and the log-rank test was performed. Cox proportional hazard model was used to analyze the independent risk factors. A difference was considered statistically significant at P < 0.05.

## Results

### Patient characteristics

Between January 2018 to December 2021, we retrospectively recruited 257 patients with advanced NSCLC, of whom 122(47.5%) advanced NSCLC patients were treated with camrelizumab plus albumin-bound paclitaxel regimen, and 135(52.5%) were camrelizumab monotherapy. Among them, 190 (73.93%) were male and 67 (26.07%) were female. Additionally, the mean age was 56.02 ± 10. 97. In addition, there were no significant differences in age, gender, treatment regimen, ECOG score, history of smoking, histopathological features, disease stage, metastases, and PD-L1 expression between CTG and STG (P>0.05), indicating that the data from CTG and STG are comparable. The details of baseline characteristics are shown in **Table 1**.

**Table 1:** Baseline characteristics of advanced NSCLC patients.

Items	All (n=257)	CTG (n=122)	STG (n=135)	X^2^/t	P
**Age**	56.02 ± 10.97	54.92 ± 11.29	56.99 ± 10.62	1.489	0.138
<65 years	76(29.6%)	33(27.00%)	43(31.9%)	0.71	0.4
Gender					
Male	190(73.9%)	93(76.2%)	97(71.9%)		
Female	67(26.1%)	29(23.8%)	38(28.1%)	0.637	0.425
treatment regimen					
Third line	148(57.6%)	64(52.5%)	84(62.2%)		
≥Third line	109(42.4%)	58(47.5%)	51(37.8%)	2.501	0.114
ECOG score, n					
0	59(23.0%)	32(26.2%)	27(20.0%)		
1	198(77.0%)	90(73.8%)	108(80.0%)	1.406	0.236
History of smoking, n					
≥20 packs a year	162(63.0%)	81(66.4%)	81(60.0%)		
<20 packs a year or never	95(37.0%)	41(33.6%)	54(40.0%)	1.124	0.289
Histopathological features					
Non-squamous carcinoma	171(66.50%)	76(62.3%)	95(70.4%)		
Squamous carcinoma	86(33.5%)	46(37.7%)	40(29.6%)	1.877	0.171
Disease stage					
IIIB–C	54(21.0%)	30(24.6%)	24(17.8%)		
IV	203(79.0%)	92(75.4%)	111(82.2%)	1.792	0.181
metastases					
Liver or Brain	84(32.7%)	43(35.2%)	41(30.4%)		
other	173(67.3%)	79(64.8%)	94(69.6%)	0.692	0.405
PD-L1 expression					
<50%	181(70.4%)	80(65.6%)	101(74.8%)		
≥50%	76(29.6%)	42(34.4%)	34(25.2%)	2.628	0.105

CTG, combined treatment group; STG, single treatment group; ECOG, Eastern Cooperative Oncology Group.

### Comparison of treatment response between CTG and STG

All patients underwent imaging examinations. As shown in **Table 2**, there were CR (n=11, 9.02%), PR (n=62, 50.08%), and SD (n=21, 17.21%) in CTG, which were significantly higher than those in STG [CR (n=6, 4.44%), PR (n=58, 42.96%), and SD (n=19, 14.07%)], while PD(n=28, 22.95%) in CTG was significantly lower than that in the STG (PD: n=52, 38.52%) (P<0.05). In addition, the ORR (n=73, 59.84 vs. n=64, 50.38%) and DCR (n=94, 77.05 vs. n=83, 65.93%) were statistically significant differences between CTG and STG (P<0.05). In general, these results suggest that the camrelizumab plus albumin-bound paclitaxel regimen was significantly more effective than camrelizumab monotherapy as a third- or later-line regimen for advanced NSCLC patients.

**Table 2:** Response of advanced NSCLC patients to camrelizumab monotherapy or camrelizumab plus albumin-bound paclitaxel regimen.

Best overall response, n (%)	CTG (n=122)	STG (n=135)	X^2^	P
CR	11(9.02%)	6(4.44%)		
PR	62(50.82%)	58(42.96%)		
SD	21(17.21%)	19(14.07%)		
PD	28(22.95%)	52(38.52%)	8.267	0.041
ORR	73(59.84%)	64(50.38%)	3.977	0.046
DCR	94(77.05%)	83(65.93%)	7.245	0.007

CTG, combined treatment group; STG, single treatment group; CR, complete remission; PR, partial remission; SD, stable disease; PD, progression of disease; ORR, objective response rate; DCR, disease control rate.

### Survival outcomes in advanced NSCLC patients

**Figure 1:**
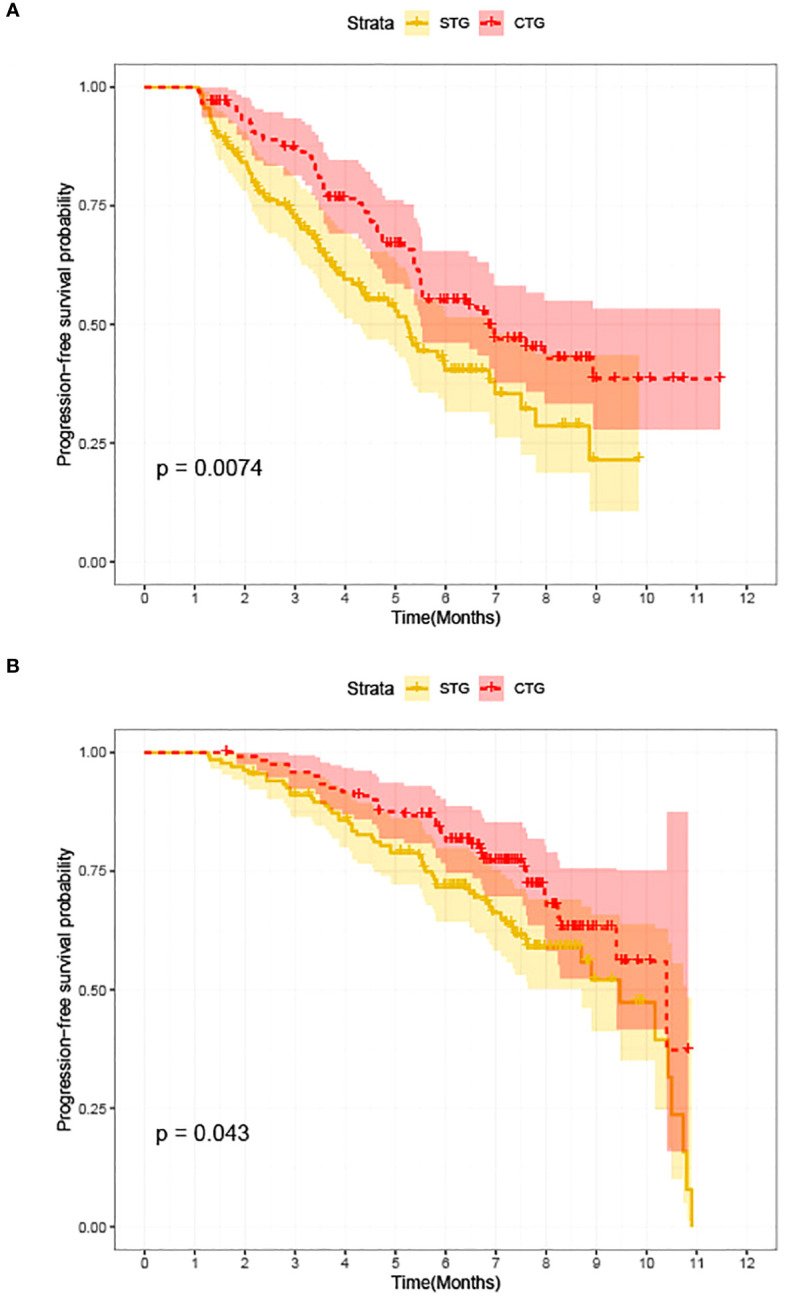
Kaplan-Meier survival between advanced NSCLC patients with camrelizumab monotherapy (STG) and camrelizumab plus albumin-bound paclitaxel regimen (CTG). **(A)** Comparison of PFS between CTG and STG; **(B)** Comparison of OS between CTG and STG.

**Figure 2:**
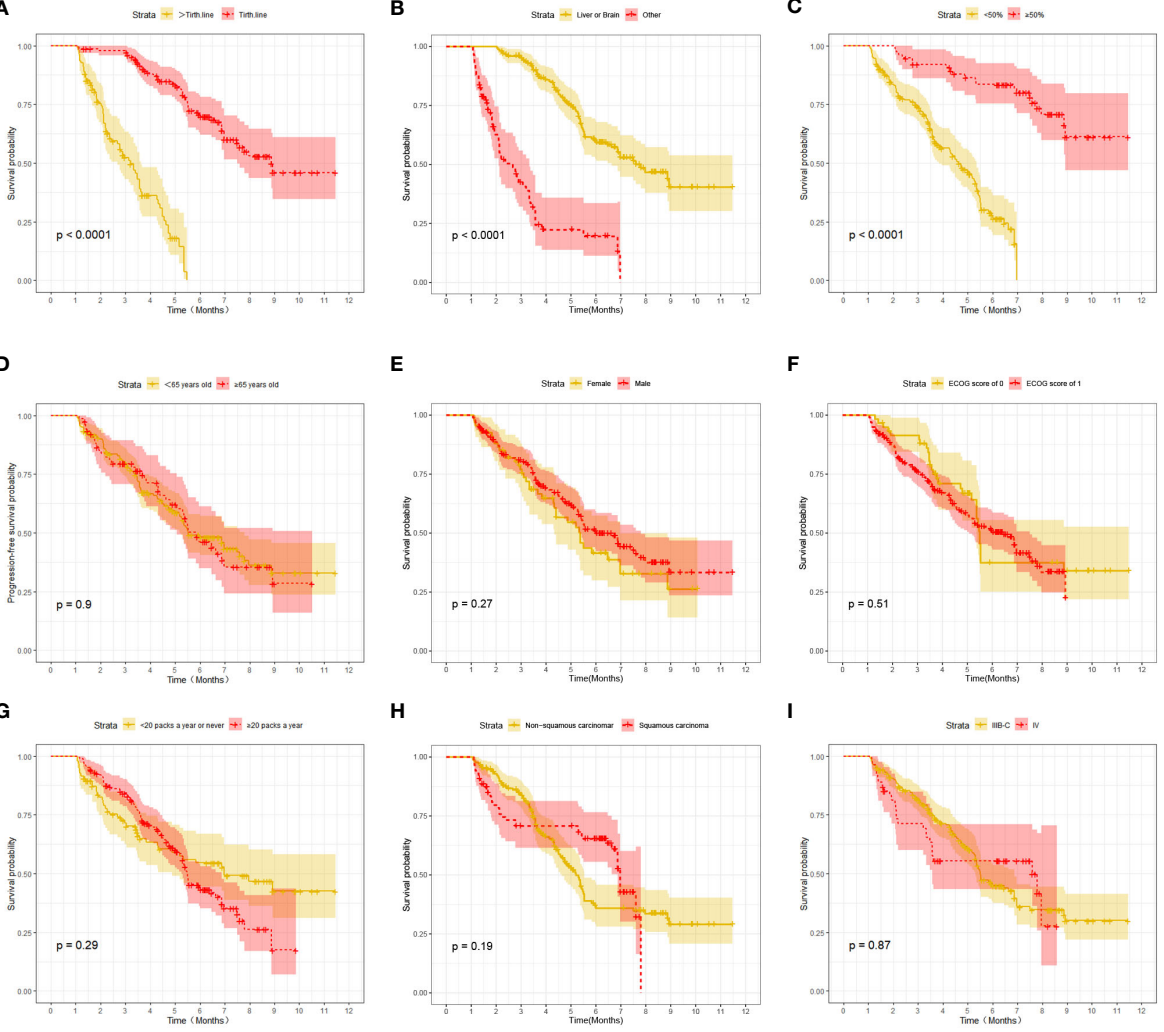
Kaplan-Meier survival (PFS) in advanced NSCLC patients between different clinical characteristics subgroups. **(A)** Comparison of PFS between Lines of treatment subgroup: third-line vs. >third-line; **(B)** Comparison of PFS between metastases subgroup: liver or brain metastasis vs. other; **(C)** Comparison of PFS between PD-L1 expression subgroup:≥50% vs. <50%; **(D)** Comparison of PFS between age subgroup: ≥65 years old vs. < 65 years old; **(E)** Comparison of PFS between gender subgroup: Male vs. Female; **(F)** Comparison of PFS between ECOG score subgroup: ECOG score of 1 vs. ECOG score of 0; **(G)** Comparison of PFS between the history of smoking subgroup: ≥20 packs a year vs. <20 packs a year; **(H)** Comparison of PFS between histopathological features subgroup: non-squamous carcinoma vs. squamous carcinoma; **(I)** Comparison of PFS between disease stage subgroup: IIIB-C vs. IV.

**Figure 3:**
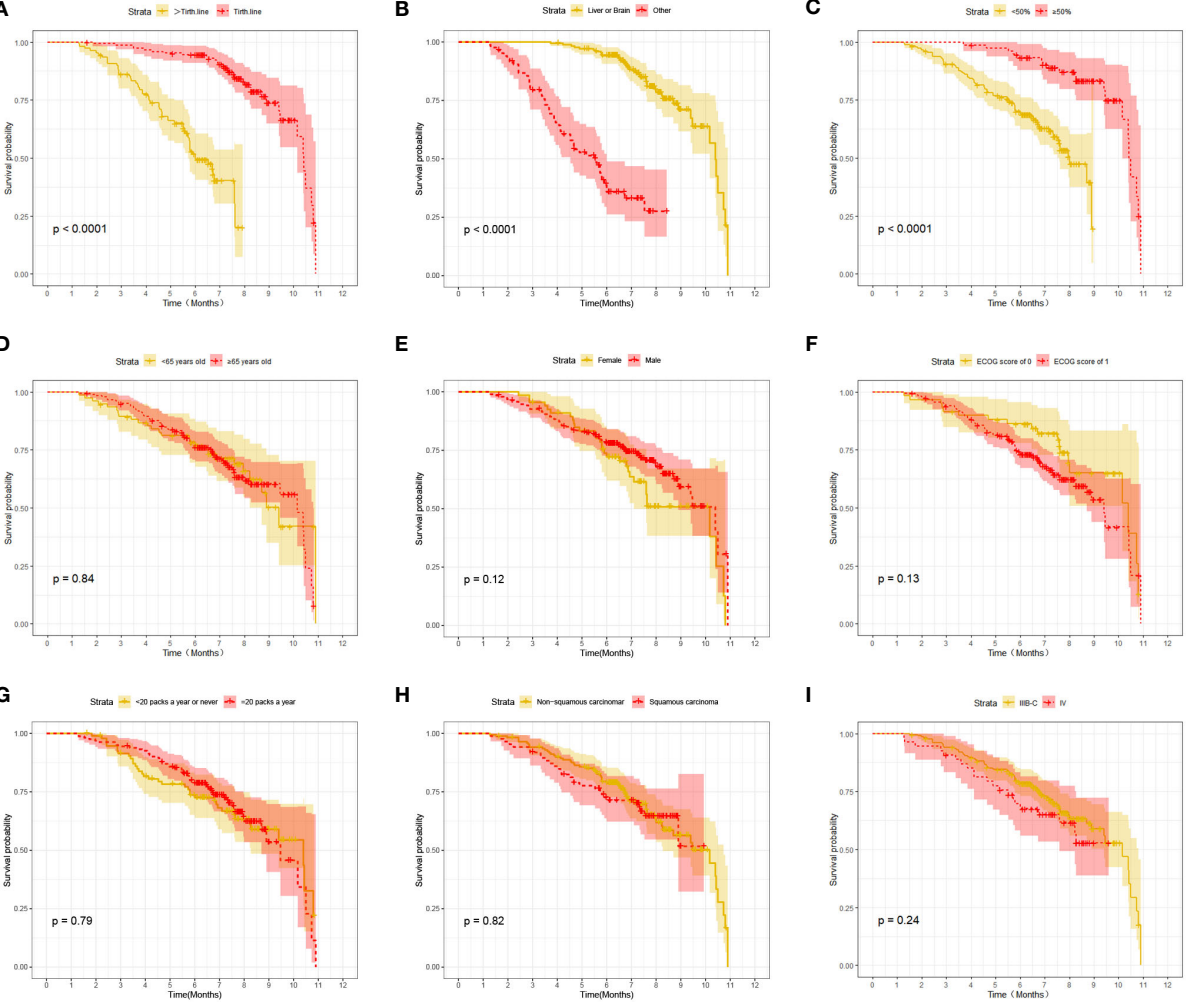
Kaplan-Meier survival (OS) in advanced NSCLC patients between different clinical characteristics subgroups. **(A)** Comparison of OS between Lines of treatment subgroup: third-line vs. >third-line; **(B)** Comparison of OS between metastases subgroup: liver or brain metastasis vs. other; **(C)** Comparison of OS between PD-L1 expression subgroup:≥50% vs. <50%; **(D)** Comparison of OSbetween age subgroup: ≥65 years old vs. < 65 years old; **(E)** Comparison of OS between gender subgroup: Male vs. Female; **(F)** Comparison of OS between ECOG score subgroup: ECOG score of 1 vs. ECOG score of 0; **(G)** Comparison of OS between the history of smoking subgroup: ≥20 packs a year vs. <20 packs a year; **(H)** Comparison of OS between histopathological features subgroup: non-squamous carcinoma vs. squamous carcinoma; **(I)** Comparison of OS between disease stage subgroup: IIIB-C vs. IV.

At the last follow-up, CTG had 28 cases of disease progression and 35 deaths recorded; while STG had 52 cases of disease progression and 57 deaths recorded. Both median PFS and median OS were longer in CTG than in STG (5.27 vs. 3.56 months, P = 0.0074 and 7.09 vs. 6.47 months, P =0.043, respectively, **Figure 1**). In addition, Compared to the >third-line group (**Figures 2A**, **3A**), liver or brain metastasis (**Figures 2B**, **3B**), and PD-L1 expression<50% (**Figures 2C**, **3C**), the third-line group, other metastasis group, and PD-L1 expression≥50% had a significantly higher median PFS (6.17 vs. 2.40 months, P<0.0001; 5.40 vs. 1.93 months, P<0.0001; 7.49 vs. 3.57 months, P<0.0001; respectively) and median OS (7.82 vs. 5.70 months, P<0.0001; 7.60 vs. 4.67 months, P<0.0001; 8.47 vs. 6.33 months, P<0.0001; respectively). However, the median PFS of the age subgroup(≥65 years old: 4.40 months vs. <65 years old: 4.33 months, P=0.9, **Figure 2D**), gender subgroup(Male: 4.60 months vs. Female: 3.97 months, P=0.27, **Figure 2E**), ECOG score subgroup(ECOG score of 1: 4.29 months vs. ECOG score of 0: 4.90 months, P=0.51, **Figure 2F**), history of smoking subgroup(≥20 packs a year or never: 4.60 months vs. <20 packs a year or never: 3.50 months, P=0.29, **Figure 2G**), histopathological features subgroup(Squamous carcinoma: 6.00 months vs. Non-squamous carcinoma: 3.97 months, P=0.19, **Figure 2H**), and disease stage subgroup(IIIB-C: 4.47 months vs. IV: 3.75 months, P=0.87, **Figure 2I**) were no significant difference. Similarly, there was no significant difference in median OS in the six subgroups (≥65 years old: 6.80 months vs. <65 years old: 6.85 months, P=0.84, **Figure 3D**; Male: 6.87 months vs. Female: 6.77 months, P=0.12, **Figure 3E**; ECOG score of 1: 6.70 months vs. ECOG score of 0: 7.57 months, P=0.13, **Figure 3F**; ≥20 packs a year or never: 6.77 months vs. <20 packs a year or never: 6.93 months, P=0.79, **Figure 3G**; Squamous carcinoma: 7.29 months vs. Non-squamous carcinoma: 6.70 months, P=0.82, **Figure 3H**; IIIB-C: 6.77 months vs. IV: 6.83 months, P=0.24, **Figure 3I**).

### Univariate and multivariate Cox proportional hazard regression analysis of factors associated with PFS and OS

As shown in **Tables 3** and **4**, in univariate analysis, lines of treatment [PFS: HR(95%CI):0.095(0.06-0.152), P=0.000; OS: HR(95%CI):0.107(0.061-0.188), P=0.000], metastases [PFS: HR(95%CI):5.917(4.057-8.629), P=0.000; OS: HR(95%CI):9.016(5.56-14.623), P=0.000], and PD-L1 expression [PFS: HR(95%CI):0.116(0.064-0.213), P=0.000; OS: HR(95%CI):0.19(0.097-0.371), P=0.000] were selected for multivariate Cox proportional hazard regression analysis (P<0.05). However, there were no significant associations between age, gender, ECOG score, history of smoking, histopathological features, and disease stage and PFS [HR(95%CI):1.001(0.985-1.017), P=0.943; HR(95%CI):0.807(0.55-1.183), P=0.271; HR(95%CI):1.149(0.759-1.739), P=0.513; HR(95%CI):1.244(0.838-1.789), P=0.296; HR(95%CI):0.774(0.525-1.141), P=0.196; HR(95%CI):0.965(0.625-1.489), P=0.872; respectively] or OS [HR(95%CI):0.999(0.981-1.018), P=0.0.946; HR(95%CI):0.708(0.458-1.094), P=0.120; HR(95%CI):1.489(0.890-2.490), P=0.129; HR(95%CI):0.943(0.617-1.442), P=0.786; HR(95%CI):1.053(0.671-1.653), P=0.823; HR(95%CI):0.868(0.510-1.479), P=0.603; respectively] in univariate analysis, as shown the **Supplementary Tables 1** and **2**. Moreover, the multivariate analysis showed that the lines of treatment [PFS: HR(95%CI):0.154(0.094-0.252), P=0.000; OS: HR(95%CI):0.184(0.099-0.344), P=0.000], metastases [PFS: HR(95%CI):3.432(2.292-5.140), P=0.000; OS: HR(95%CI):5.054(3.019-8.458), P=0.000], and PD-L1 expression [PFS: HR(95%CI):0.277(0.144-0.531), P=0.000; OS: HR(95%CI):0.458(0.22-0.954), P=0.037] were the independent risk factors for the PFS and OS of patients with advanced NSCLC.

**Table 3:** Univariate and Multivariate Cox proportional hazard regression analysis of factors associated with PFS.

	Univariate Cox Regression			P	Multivariate Cox Regression			P
	HR	95% CI			HR	95% CI		
		down	upper			down	upper	
Lines of treatment (Third-line/>Third-line)	0.095	0.06	0.152	0.000	0.154	0.094	0.252	0.000
metastases (Liver or Brain/Other)	6	4.11	8.76	0.000	3.432	2.292	5.14	0.000
PD-L1 expression (≥50%/<50%)	0.112	0.061	0.208	0.000	0.277	0.144	0.531	0.000

CTG, combined treatment group; STG, single treatment group.

**Table 4:** Univariate and Multivariate Cox proportional hazard regression analysis of factors associated with OS.

	Univariate Cox Regression			P	Multivariate Cox Regression			P
	HR	95% CI			HR	95% CI		
		down	upper			down	upper	
Lines of treatment (Third-line/>Third-line)	0.107	0.061	0.188	0.000	0.184	0.099	0.344	0.000
metastases (Liver or Brain/Other)	9.016	5.56	14.623	0.000	5.054	3.019	8.458	0.000
PD-L1 expression (≥50%/<50%)	0.19	0.097	0.371	0.000	0.458	0.22	0.954	0.037

CTG, combined treatment group; STG, single treatment group.

### Safety

A total of 257 advanced NSCLC patients were systemically assessed for safety, as shown in **Table 5**. No fatal adverse events (AEs) were observed and most AEs were mild and manageable. In addition, the common AEs in camrelizumab monotherapy or camrelizumab plus albumin-bound paclitaxel regimen are reactive cutaneous capillary endothelial proliferation (RCCEP) (79.26% and 80.33%, respectively), followed by hematologic toxicity, including neutrophil count decreased (69.67% and 65.19%, respectively), white blood cell count decreased (68.03% and 53.33%, respectively), anemia (62.30%% and 62.22%, respectively), and platelet count decreased (48.36% and 39.26%, respectively). Additionally, grade (≥3) toxicity-related events were neutrophil count decreased (34.43% and 28.15%, respectively), white blood cell count decreased (38.52% and 13.33%, respectively), anemia (17.21% and 8.15%, respectively), and platelet count decreased (17.21% and 11.11%, respectively). These results suggest similar AEs between CTG and STG. Importantly, None of the patients delayed treatment because of the AEs of the regimen.

**Table 5:** Adverse events in CTG and STG.

Haematological toxicities	CTG (n=122)		STG (n=135)	
	All Grades	Grade ≥3	All Grades	Grade ≥3
Neutrophil count decreased	85(69.67%)	42(34.43%)	88(65.19%)	38(28.15%)
White blood cell count decreased	83(68.03)	47(38.52%)	72(53.33%)	18(13.33%)
Anaemia	76(62.30%)	21(17.21%)	84(62.22%)	11(8.15%)
Platelet count decreased	59(48.36%)	21(17.21%)	53(39.26%)	15(11.11%)
Lymphocyte count decreased	15(12.30%)	6(4.92%)	12(8.89%)	3(2.22%)
Haemoglobin decreased	13(10.66%)	1(<1%)	11(8.15%)	0
Non-haematological toxicities				
Reactive cutaneous capillary endothelial proliferation	98(80.33%)	1(<1%)	107(79.26%)	0
Aspartate aminotransferase increased	54(44.26%)	1(<1%)	58(42.96%)	0
Alanine aminotransferase increased	49(40.16%)	4(3.28%)	51(37.78%)	1(<1%)
Nausea	46(37.70%)	1(<1%)	47(34.81%)	0
Asthenia	39(31.97%)	4(3.28%)	39(28.89%)	2(1.48%)
Decreased appetite	38(31.15%)	4(3.28%)	36(26.67%)	3(2.22%)
Constipation	27(22.13%)	0	26(19.26%)	0
Vomiting	26(21.31%)	1(<1%)	27(20.00%)	1(<1%)
Hepatic function abnormal	27(22.13%)	4(3.28%)	28(20.74%)	2(1.48%)
Gamma-glutamyltransferase increased	23(18.85%)	5(4.10%)	20(14.81%)	3(2.22%)
Rash	16(13.11%)	2(1.64%)	16(45.71%)	1(<1%)
Pruritus	16(13.11%)	1(<1%)	15(11.11%)	0
Blood creatinine increased	12(9.84%)	0	11(8.15%)	1(<1%)
Hypothyroidism	15(12.30%)	1(<1%)	15(11.11%)	0
Blood bilirubin increased	16(13.11%)	0.00	16(11.85%)	1(<1%)

CTG, combined treatment group; STG, single treatment group.

## Discussion

Previous studies have shown that ICIs directly restore the tumor-mediated depleted host anti-tumor immune response compared to other anti-tumor treatments such as chemotherapy, radiotherapy, and targeted therapy (9). At present, ICIs have been approved for various malignancies treatment, such as head-neck malignant tumor and melanoma (10). Over the past decades, Immune checkpoint blockade targeting PD-1/PD-L1 treatment has emerged as a first- or second-line treatment standard for advanced NSCLC patients, because of its significant therapeutic efficacy. Literature has shown that anti-PD1/PD-L1 immunotherapy increases the 5-year survival rate of advanced NSCLC patients from <5% to 26% (11, 12). Camrelizumab, as the first domestic anti-tumor PD-1 antibody, specifically targets PD-1 and blocks its binding to PD-L1 and programmed death ligand 2 (PD-L2), thereby restoring the body’s immune function and ultimately exerting anti-tumor effects.

Currently, the applications of camrelizumab include relapsed or refractory classic Hodgkin’s lymphoma (13), advanced hepatocellular carcinoma (14), and advanced esophageal squamous carcinoma (15). In addition, camrelizumab in combination with pemetrexed and carboplatin regimen is the first-line regimen for non-squamous NSCLC patients (7). In addition, many clinical studies also showed other applications of camrelizumab for advanced NSCLC patients. Yinhua Wang’s (16) and Guanghui Gao’s (17) studies suggested that camrelizumab plus vascular endothelial growth factor(VEGF) indicators significantly prolonged PFS and OS of advanced NSCLC patients. Similarly, camrelizumab plus microwave ablation also showed significant efficacy in the advanced NSCLC patients with a median of PFS 11.8 months (18).

Despite the increasingly important role of PD-1 in the treatment of NSCLC, a study showed that a small proportion of NSCLC patients do not benefit from immunotherapy monotherapy, and chemotherapy remains the main treatment in these patients (19). Paclitaxel is a classical anti-tumor drug that has played a significant role in the treatment of various malignancies such as lung, ovarian, and breast cancers (20). Albumin-bound paclitaxel forms a novel nanoparticle formulation with albumin as a carrier to circumvent the problem that paclitaxel itself is extremely insoluble in water (21), which significantly reduces the allergic reactions caused by paclitaxel with a higher concentration in tumor tissues, therefore bringing more clinical efficacy and less toxic side effects to cancer patients (22).

Hence, in this study, we proposed a third- or later-line regimen of camrelizumab plus albumin-bound paclitaxel for advanced NSCLC patients. The current study showed that the ORR and DCR in CTG were significantly higher than those in STG. Importantly, the median PFS (5.27 vs. 3.57 months) and median OS (7.09 vs. 6.47 months) in the CTG were significantly longer than those in the STG. These results suggest that immunotherapy plus chemotherapy was an ideal treatment option for tolerable patients with advanced NSCLC (23, 24). In addition, the median PFS of 5.27 months in CTG was shorter than the previous studies (camrelizumab or protein-bound paclitaxel as first-line regimen: 8.3 months and 6.3 months, respectively) (7, 22), and similar to Caicun Zhao’s research (5.7 months) (25). Furthermore, the median OS of 7.09 in CTG was lower than camrelizumab combined with chemotherapy as the second-line regimen (8). However, the ORR and DCR in our study were higher than in these studies (7, 22, 25). As for camrelizumab monotherapy, our results showed better median PFS (3.57 months) and median OS (6.47 months) than those in the previous literature(median PFS of 2.8 months, and median OS not reached) (26). Overall, a third- or later-line regimen of camrelizumab plus albumin-bound paclitaxel greatly prolonged PFS and OS in patients with advanced NSCLC, suggesting that the regimen in our study has a better trend for survival for advanced NSCLC patients compared to camrelizumab monotherapy. which provide a third- or later-line treatment regimen for advanced NSCLC patients in clinical practice.

Moreover, Our study also found third-line treatment, other metastases, and PD-L1 expression ≥50% were found to be associated with prolonged PFS and OS, which was consistent with the results in the Cox proportional hazard regression. Importantly, Cox proportional hazard regression further identified that the > third-line, liver or brain metastasis, and PD-L1 expression <50% were the independent risk factors for the median PFS and median OS in advanced NSCLC patients, which was consistent with previous studies (8, 27, 28).

Although ICIs have prolonged the survival time of malignant patients, immune-related adverse events (irAEs) usually involve multiple body systems such as the skin, endocrine organs, and liver (29). Reactive cutaneous capillary endothelial proliferation (RCCED), a type of irAEs occurring mainly in the skin and is characterized by increased capillaries in the dermis and proliferation of capillary endothelial cells, is the most common drug-related adverse reaction of carlinibizumab. Previous studies have shown that the incidence of REECD ranged from 66.8% to 97.3% with carlinibizumab monotherapy (15, 30–32), which is consistent with our study’s findings. In addition, grade (≥3) toxicity-related events occurs in this study, but no fatal adverse events (AEs) were observed and most AEs were mild and manageable. Most symptoms recovered on their own after a period of discontinuation of the drug. These results suggest that the toxicity of the carlinibizumab plus albumin-bound paclitaxel regimen was tolerable, and this regimen could be used in clinical practice for advanced NSCLC in the future.

However, our study had some limitations. First, this study was based on retrospective data, and a selection bias may have existed. Therefore, forward-looking external validation is required. At present, there are not many effective treatment methods for the patients with advanced NSCLC who have failed second line regimen. The present study provides a potential regimen. Hence, the future research should focused on the patients with advanced NSCLC, and multiple endpoints such as OS, PFS, ORR, DCR, et al.

## Conclusion

The treatment regimen of camrelizumab plus albumin-bound paclitaxel has promising efficacy and manageable toxic effects as a third- or later-line treatment for patients with advanced NSCLC. The prognosis of patients with advanced NSCLC may be related to the type of metastasis, the lines of treatment, and PD-L1 expression levels. However, the clinical efficacy of carlinibizumab in combination with albumin-bound paclitaxel as a third- or later-line regimen for patients with advanced NSCLC needs to be further confirmed due to the retrospective bias of this study. PD-L1 expression level, driver gene status, liver or brain metastasis, and the economic burden of patients with advanced NSCLC should be taken into consideration for clinical decision-making.

## Data availability statement

The original contributions presented in the study are included in the article/**Supplementary Material**. Further inquiries can be directed to the corresponding author.

## Ethics statement

The studies involving humans were approved by The Ethics Committee of Jiangxi Province Cancer hospital. The studies were conducted in accordance with the local legislation and institutional requirements. The ethics committee/institutional review board waived the requirement of written informed consent for participation from the participants or the participants’ legal guardians/next of kin because no personally identifiable information was provided.

## Author contributions

JZ: Conceptualization, Methodology, Writing – original draft. YY: Formal analysis, Software, Validation, Writing – review & editing. JM: Data curation, Project administration, Resources, Writing – review & editing. SC: Data curation, Formal analysis, Writing – review & editing. JL: Formal analysis, Investigation, Software, Writing – review & editing. SJ: Conceptualization, Data curation, Formal analysis, Methodology, Writing – original draft.
