# Supplementary material for: Efficacy and safety of camrelizumab combined with albumin-bound paclitaxel as third- or later-line regimen in patients with advanced non-small cell lung cancer
Source: Front Immunol. 2023 Dec 6;14:1278573. doi: 10.3389/fimmu.2023.1278573 (PMC10731289; doi:10.3389/fimmu.2023.1278573)
Supplement: Supplementary file 1 [file Table_1.docx]

**Supplementary Table 1** Univariate Cox proportional hazard regression analysis of factors associated with PFS

| Clinical characteristics | HR | 95% CI | | P |
| --- | --- | --- | --- | --- |
|  |  | down | upper |  |
| Age | 1.001 | 0.985 | 1.017 | 0.943 |
| Gender | 0.807 | 0.55 | 1.183 | 0.271 |
| ECOG score | 1.149 | 0.759 | 1.739 | 0.513 |
| History of smoking | 1.224 | 0.838 | 1.789 | 0.296 |
| Histopathological features | 0.774 | 0.525 | 1.141 | 0.196 |
| Disease stage | 0.965 | 0.625 | 1.489 | 0.872 |

**Supplementary Table 2** Univariate Cox proportional hazard regression analysis of factors associated with OS

| Clinical characteristic | HR | 95% CI | | P |
| --- | --- | --- | --- | --- |
|  |  | down | upper |  |
| Age | 0.999 | 0.981 | 1.018 | 0.946 |
| Gender | 0.708 | 0.458 | 1.094 | 0.12 |
| ECOG score | 1.489 | 0.89 | 2.49 | 0.129 |
| History of smoking | 0.943 | 0.617 | 1.442 | 0.786 |
| Histopathological features | 1.053 | 0.671 | 1.653 | 0.823 |
| Disease stage | 0.868 | 0.51 | 1.479 | 0.603 |
